# A Rare Case Report of Immunoglobulin D Subtype Multiple Myeloma Complicated With Hyperlipidemia

**DOI:** 10.1155/crh/9065226

**Published:** 2026-04-17

**Authors:** Liping Yang, Zhimei Zhou, Xiaoyuan Qu, Lili Jing, Juanjuan Liu, Li Chen, Linfeng Ma

**Affiliations:** ^1^ Department of Clinical Laboratory, Qingdao University Medical College Affilited Yantai Yuhuangding Hospital, Yantai, 264099, China; ^2^ Department of Medicine, Shandong College of Traditional Chinese Medicine, Yantai, 264199, China

## Abstract

Multiple myeloma is a plasma cell malignancy characterized by complex heterogeneous cytogenetic abnormalities. In most cases, patients suffering from multiple myeloma typically display normal or decreased blood lipid concentrations. We herein present a case of immunoglobulin (Ig) D‐λ subtype multiple myeloma with concurrent hyperlipidemia. This patient presented with elevated lipid levels upon lenalidomide, bortezomib, and dexamethasone (RVD) chemotherapy with triglycerides of 4.12 mmol/L (reference range: 0.4–1.7 mmol/L), total cholesterol of 6.71 mmol/L (reference range: 3.12–5.72 mmol/L), low‐density lipoprotein cholesterol of 4.29 mmol/L (reference range: 1.04–1.96 mmol/L), and lipoprotein(a) of 582 mg/L (reference range: 0–300 mg/L), which showed typical symptoms of hyperlipidemia. After chemotherapy cycles, the elevated lipid indicators were gradually decreased (or close) to normal levels without any antihyperlipidemia drug or therapy. This report analyzed a rare hyperlipidemic multiple myeloma case and may provide insights into the safety and guidance of the procedure for clinical diagnosis and treatment.

## 1. Introduction

Multiple myeloma is characterized by the uncontrolled proliferation of clonal malignant plasma cells in the bone marrow and accompanied by an elevated single clone of immunoglobulin (Ig) in the serum or urine [1]. The clinical presentation generally manifests as hypercalcemia (C), renal failure (R), anemia (A), bone pain, and pathological fractures (B) [2]. In the majority of patients with multiple myeloma, blood lipid concentrations are usually decreased or within the normal range [3]. However, a limited number of cases indicated that the significant presentation of M protein may be associated with marked elevations in serum triglycerides and cholesterol, especially in patients diagnosed with IgA‐ and IgG‐type multiple myeloma [3, 4]. IgD multiple myeloma is a rare subtype that affects 1%–2% of all patients with multiple myeloma abroad, while 3%–8.9% are affected in domestic cases [5]. Clinically, IgD‐type multiple myeloma is frequently associated with more aggressive disease symptoms and manifests high‐risk features (such as severe complications and comorbidities, younger age, high incidence of extramedullary infiltration, bone destruction, hypercalcemia, renal insufficiency, and genetic abnormalities) with inferior survival [6]. However, IgD‐type multiple myeloma has rarely been reported to be associated with hyperlipidemia.

Here, we report a rare hyperlipidemic case of a 59‐year‐old male patient with IgD‐type multiple myeloma. The patient manifested the symptoms of hyperlipidemia during lenalidomide, bortezomib, and dexamethasone (RVD) treatment. The elevated lipid indicators were gradually decreased (or close) to normal levels after chemotherapy without any antihyperlipidemia drug or therapy.

## 2. Case Presentation

A 59‐year‐old Chinese male patient was admitted to our hospital with thoracalgia, paresthesia, and intermittent claudication on April 6, 2021. Physical examination showed tenderness in the mid‐to‐lower thoracic spinous process spaces and paraspinal soft tissues. No masses were palpable in the bilateral buttocks, and there was no deep tenderness in the sciatic nerve exit. The muscle strength of both lower limbs was Grade 4–5. He had a past medical history of a left leg fracture in 2017 but no history of hyperlipidemia, hypertension, cardiopathy, diabetes mellitus, cardiovascular disease, or psychiatric disorders.

On admission, initial laboratory tests revealed anemia, leukocytosis, and abnormal renal function with a red blood count of 2.48 × 10^12^/L, a hemoglobin level of 75 g/L, and a white blood cell count of 11.23 × 10^9^/L (Table 1). Meanwhile, the concentrations of serum light chain λ and urine light chain λ were 1430 mg/L and 2270 mg/L, respectively. Serum protein electrophoresis displayed a markedly abnormal appearance of a monoclonal M‐spike (Figure 1(a)). Besides, 28.2% abnormal plasma cells of karyocytes and 3.8% Igλ plasma cells of total cells were detected by multicolor flow cytometry (Figure 1(b)). He was given a diagnosis of the IgD‐λ subtype of multiple myeloma (Stage III B). However, he had no signs or symptoms of hyperlipidemia (with normal concentrations of TG, TC, HDL, LDL, and LPa; Table 1) at this stage.

**TABLE 1:** Laboratory data on admission.

Laboratory data	Result	Reference ranges
RBC (10^12^/L)	**2.48**	4.3–5.8
HGB (g/L)	**75**	130–175
WBC (10^9^/L)	**11.23**	3.5–9.5
PLT (10^9^/L)	224	125–350
Urine protein (mg/L)	**3.84**	0–0.15
UA (μmol/L)	**691**	208–428
BUN (mmol/L)	**14.71**	3.1–8
Crea (μmol/L)	**304**	57–97
GLU (mmol/L)	5.02	3.8–6.2
Ca (μmol/L)	**2.89**	2.11–2.52
TBil (μmol/L)	11.9	3.2–23.5
DBiL (μmol/L)	1.7	0–6.8
GGT (U/L)	42	10–60
ALT (U/L)	48	9–50
AST (U/L)	39	15–40
LDH (U/L)	200	120–250
NT‐proBNP (mg/L)	**1068**	0–450
CRP (mg/L)	3.22	0–6
Fe (μmol/L)	20.1	10.6–36.7
TBIL (μmol/L)	62.1	45–75
UBIL (μmol/L)	42	31–51
TP (g/L)	68.01	65–85
ALB (g/L)	**34.61**	40–55
GLO (g/L)	**43.55**	20–40
Albumin/globulin	**0.795**	1.2–2.4
Serum IgG (mg/dL)	5.09	7–16
Serum IgA (mg/dL)	0.35	0.7–4
Serum IgM (mg/dL)	0.166	0.4–2.3
Serum LC κ (mg/L)	1.99	1.7–3.7
Serum LC λ (mg/L)	**1430**	0.9–2.1
Serum LC *κ*/*λ*	**0.001**	0.26–1.65
Urine LC κ (mg/L)	**13.5**	0–7.44
Urine LC λ (mg/L)	**2270**	0–4.09
Urine LC *κ*/*λ*	**0.006**	0.15–1.85
β2‐MG (mg/L)	**6.3**	1.09–2.53
TG (mmol/L)	1.44	0.4–1.7
TC (mmol/L)	2.68	3.12–5.72
HDL (mmol/L)	0.69	1.04–1.96
LDL (mmol/L)	1.48	1.53–3.45
LPa (mg/L)	239	0–300

*Note:* The significance of bold data indicates that values are either above or below the normal range.

Abbreviations: Ig, immunoglobulin; LC, light chain.

FIGURE 1Serum protein electrophoresis (a) and multicolor flow cytometry (b) analysis before RVD chemotherapy.

**Figure:**
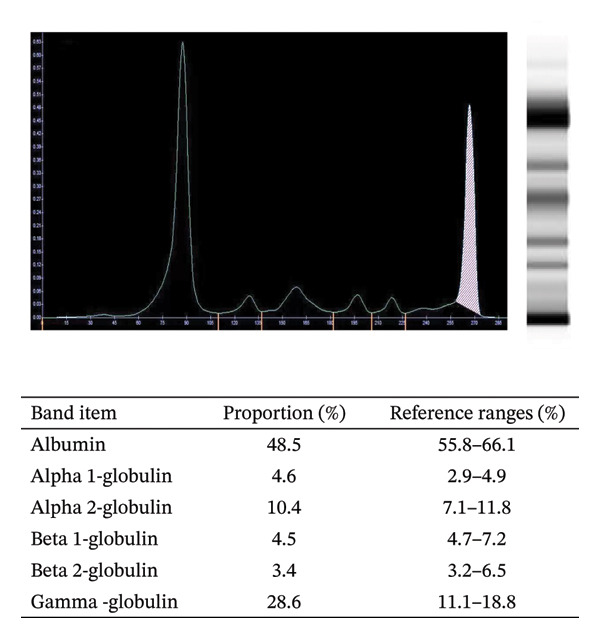
(a)

**Figure:**
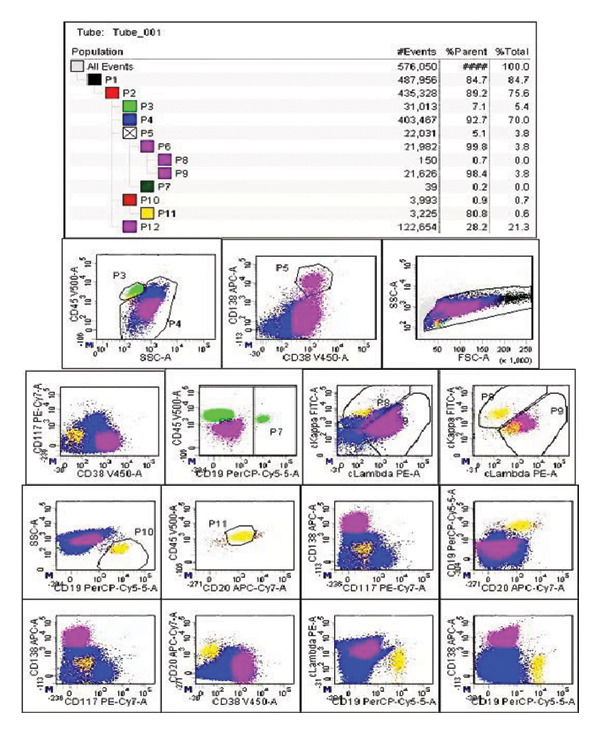
(b)

**FIGURE 3:**
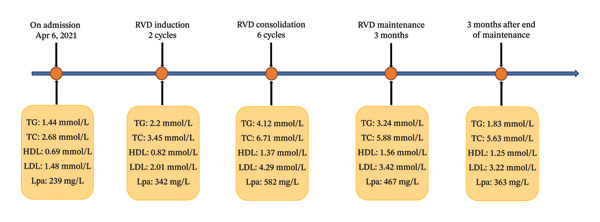
Clinical course and serum lipid indicators of the patient. The red font indicates that the data exceed the upper limit of reference. Reference ranges: TG, 0.4–1.7 mmol/L; TC, 3.12–5.72 mmol/L; HDL, 1.04–1.96 mmol/L; LDL, 1.53–3.45 mmol/L; LPa, 0–300 mg/L.

This patient received RVD induction treatment with oral lenalidomide (25 mg daily on Days 1–14), subcutaneous bortezomib (1.3 mg/m^2^ on Days 1, 4, 8, and 11), and oral dexamethasone (20 mg on Days 1, 2, 8, 9, 15, and 16). After two RVD induction cycles, the concentrations of serum light chain λ and urine light chain λ were less than 7.13 mg/L and 66.9 mg/L, respectively. Moreover, not only the area of monoclonal M‐spike was significantly decreased (less than 1%; Figure 2(a)), but also the percentage of Igλ plasma cells was decreased to less than 0.1% of total cells (Figure 2(b)), suggesting that this patient achieved a good response to RVD chemotherapy. However, the serum levels of TG (2.2 mmol/L, normal: 0.4–1.7 mmol/L) and LPa (342 mg/L, normal: 0–300 mg/L) exceeded the reference ranges (Figure 3).

FIGURE 2Serum protein electrophoresis (a) and multicolor flow cytometry (b) analysis after RVD chemotherapy.

**Figure:**
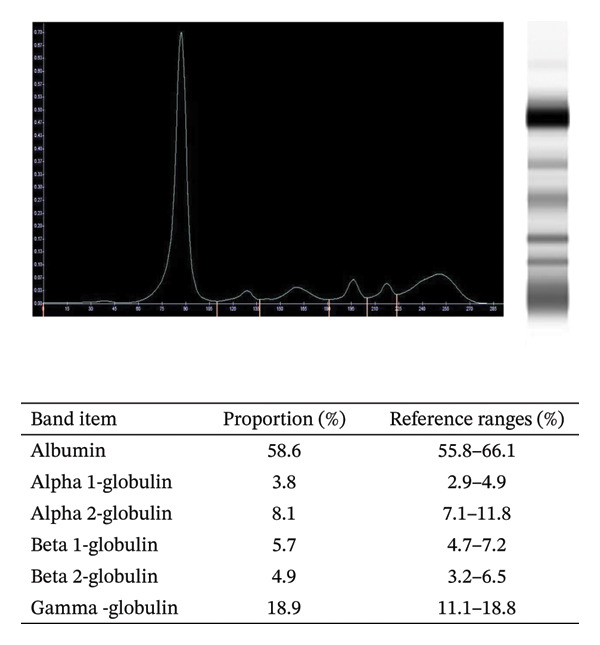
(a)

**Figure:**
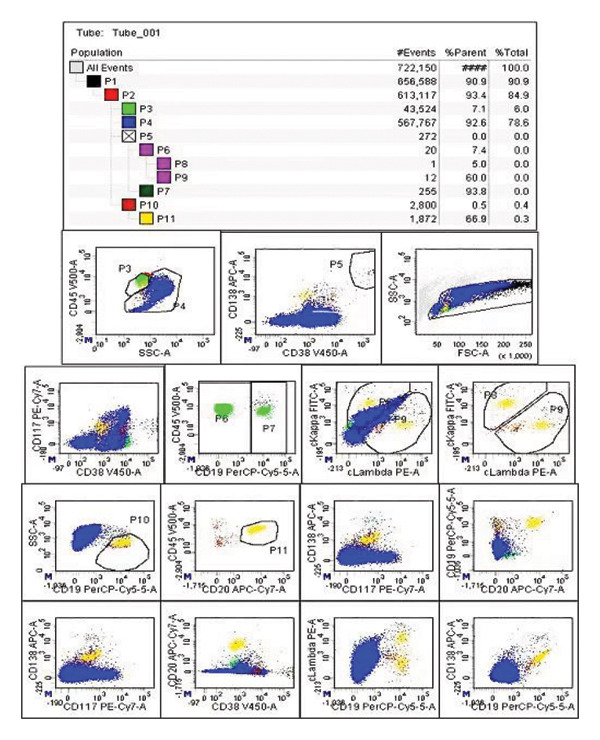
(b)

Then, he underwent six RVD consolidation cycles (21 days for each cycle, 168 days in total). After RVD consolidation of six cycles, serum and urine light chain λ were negative. However, to our surprise, the serum concentrations of TG, TC, LDL‐C, and LPa were continuously increased to 4.12 mmol/L, 6.71 mmol/L, 4.29 mmol/L, and 582 mg/L (approximately 2.9‐, 2.5‐, 2.9‐, and 2.4‐fold higher than baseline), which displayed typical characteristics of hyperlipidemia (Figure 3).

A subsequent bortezomib and dexamethasone maintenance therapy (one dose of 1.3 mg/m^2^ bortezomib and two doses​ of 20 mg dexamethasone every week) was conducted for 3 months. In this stage, the serum concentration of LDL has reached the normal range. Besides, the levels of TG (3.24 mmol/L), TC (5.88 mmol/L), and LPa (467 mg/L, Figure 3) showed a slight decrease when compared to the RVD consolidation stage. Next, 3 months after the end of chemotherapy cycles, the elevated lipid indicators were gradually decreased (or close) to normal levels (TG 1.83 mmol/L, TC 5.63 mmol/L, and LDL‐C 3.22 mmol/L; Figure 3) without any antihyperlipidemia drug or therapy. At 3‐month follow‐up, he remained well, and no adverse reactions occurred. In addition, considering that the patient’s body mass index (BMI) is normal and that there was no family history of hyperlipidemia, this patient did not receive further medications.

## 3. Discussion

Hyperlipidemia is an uncommon and poorly understood symptom of multiple myeloma. In a retrospective study by Misselwitz et al., 53 patients with multiple myeloma met the criteria for hyperlipidemia, and most of them were diagnosed with IgA‐ and IgG‐type multiple myeloma [4]. However, IgD‐type multiple myeloma has been rarely reported and remains poorly understood. In this case, the patient initially exhibited renal impairment (with uric acid of 694 μmol/L, urea of 14.71 mmol/L, and creatinine of 304 μmol/L) and inflammation (serum light chain *λ*: 1430 mg/L; urine light chain *λ*: 2270 mg/L) on admission. It is well known that renal impairment and inflammation may be involved in the pathogenesis of hyperlipidemia [7, 8]. However, the patient displayed a normal lipid profile upon admission, which indicated that hyperlipidemia may not be secondary to kidney disease or inflammation.

In this case, the elevated lipid levels were identified during RVD treatment (especially in the RVD consolidation stage), with TG of 4.12 mmol/L (reference range: 0.4–1.7 mmol/L), TC of 6.71 mmol/L (reference range: 3.12–5.72 mmol/L), LDL of 4.29 mmol/L (reference range: 1.04–1.96 mmol/L), and LPa of 582 mg/L (reference range: 0–300 mg/L). RVD treatment is a standard frontline therapy for patients with newly diagnosed multiple myeloma (NDMM) [9]. This regimen demonstrated remarkable efficacy with a stringent complete response (sCR) in 51% of patients, achieving at least a very good partial response (VGPR) in 96% as the best response [10]. However, hyperlipidemia was previously recorded as an adverse event in patients treated with bortezomib and dexamethasone [11]. Moreover, after chemotherapy cycles, the elevated lipid indicators were gradually decreased (or close) to normal levels without any antihyperlipidemia drug or therapy. Hence, we hypothesize that the process of chemotherapy may contribute to the development of hyperlipidemia in this patient.

Bortezomib, a proteasome inhibitor, is widely used for the treatment of multiple myeloma due to its ability to induce cell cycle arrest and trigger apoptosis [12]. Emerging evidence indicates that bortezomib modulates lipid metabolism by upregulating fatty acid synthase (FAS) inhibition, promoting the buildup of ubiquitinated proteins, and stimulating de novo fatty acid production [13]. Besides, dexamethasone is a long‐acting anti‐inflammatory synthetic steroid and well known to cause hyperlipidemia, lipid peroxidation, and hematological alterations [14]. Importantly, dexamethasone was confirmed to provoke a significant elevation in serum levels of TC, TG, LDL, and HDL [15]. Mechanistically, dexamethasone exposure disrupts lipid metabolism by regulating the sterol regulatory element binding transcription factor 1 (SREBP‐1), acyl‐CoA dehydrogenase long chain (ACADL), and acyl‐CoA synthetase bubblegum family member 1 (ACSBG1) genes [16]. Moreover, a previous study indicated that lenalidomide upregulates cholesterol synthesis followed by an enhanced rate of cell cycle and cholesterol biosynthesis [17]. In this case, the patient presented with elevated lipid levels upon RVD chemotherapy with TG of 4.12 mmol/L, TC of 6.71 mmol/L, LDL of 4.29 mmol/L, and LPa of 582 mg/L, which showed typical symptoms of hyperlipidemia. Coincidentally, a previous hyperlipidemic case was reported by Gotoh et al., in which they described a patient with multiple myeloma who developed elevated TC and TG levels after two courses of bortezomib and dexamethasone treatment [11]. Therefore, we hypothesize that RVD administration may be an inducer in the pathogenesis of hyperlipidemia.

Next, the bone marrow microenvironment has a crucial role in the multiple myeloma pathogenesis and significantly contributes to systemic dysregulation of lipid metabolism in plasma cell malignancies [18]. Altered lipid metabolism may intersect with mechanisms of drug resistance and radioresistance in several solid tumor cancer types as well as in multiple myeloma [19, 20]. For example, sphingolipids have been recognized as mediators of the immune response and drug resistance in multiple myeloma [21]. Importantly, previous studies demonstrated that bortezomib and dexamethasone treatment disrupts the hemostasis of lipid profiles, leading specifically to aberrant sphingolipid metabolism [22, 23]. Thus, bortezomib and dexamethasone may be associated with the changes in the multiple myeloma microenvironment and intersect with dysregulation of lipid profiles. Further studies and clinical validation are required to verify these findings and explore the possible mechanisms.

Finally, the limitation of this case report should also be considered. First, we did not monitor the changes in blood concentrations of bortezomib, dexamethasone, and lenalidomide in this patient. This deficiency cuts the possibility to demonstrate a direct correlation between RVD treatment and hyperlipidemia. Second, this report lacks the perspective of the patient and misses the opportunity to present patient‐centered reporting.

## Author Contributions

Liping Yang and Linfeng Ma conceptualized the case report. Liping Yang, Zhimei Zhou, and Linfeng Ma reviewed and confirmed the patient data. Xiaoyuan Qu, Lili Jing, Juanjuan Liu, Li Chen, and Linfeng Ma conducted the literature review. All authors wrote and reviewed the final manuscript.

## Funding

This study was not supported by any funder or sponsor.

## Disclosure

All authors have read and approved the final version of the manuscript. Linfeng Ma had full access to all of the data in this study and takes complete responsibility for the integrity of the data and the accuracy of the data analysis.

## Ethics Statement

Informed consent and data analysis protocols were obtained through the approved means of the hospital’s ethics guidelines.

## Consent

The patient has signed the consent statement (No. 2202337), and all identifying details were removed to protect confidentiality.

## Conflicts of Interest

The authors declare no conflicts of interest.

## Data Availability

The data that support the findings of this study are available on request from the corresponding author upon reasonable request. The data are not publicly available due to privacy or ethical restrictions.
